# Short and long-term outcome of levamisole in early versus late steroid responsive nephrotic syndrome: A single centre experience

**DOI:** 10.12669/pjms.41.1.10184

**Published:** 2025-01

**Authors:** Saima Kashif, Khemchand N Moorani

**Affiliations:** 1 Saima Kashif, MBBS, FCPS (Pediatrics), FCPS Pediatr Nephrol. Assistant Professor, Department of Pediatric Nephrology, The Kidney Centre Post Graduate Training Institute, Karachi. 197/9, Rafiqui Shaheed Road, Karachi-75530, Pakistan; 2 Khemchand N Moorani, MBBS, MCPS, FCPS, IPNA Pediatr Nephrol fellowship Professor, Department of Pediatric Nephrology, The Kidney Centre Post Graduate Training Institute, Karachi. 197/9, Rafiqui Shaheed Road, Karachi-75530, Pakistan

**Keywords:** Levamisole, Outcome, Steroid responsive nephrotic syndrome, Children

## Abstract

**Objectives:**

To determine the effectiveness of Levamisole (Leva) in maintaining short-term and long-term remission in early steroid responders (ESRs) and late steroid responders (LSRs).

**Methods:**

This retrospective study on 106 cohorts, aged 2-14 years with frequent-relapsing (FR) and steroid-dependent nephrotic syndrome (SDNS) who received Leva over 10-years (2012-2023), was carried out at tertiary care centre, Karachi from January-August 2023. Patients were categorized based on steroid response during first episode of NS as ESRs if complete remission (CR) was achieved within two weeks of daily steroid and LSRs if CR achieved between two-four weeks. Leva was administered after inducing remission with daily steroid after labelling as FRNS and SDNS. Low-dose steroid was continued during Leva-therapy. Short-term outcome was assessed at completion of Leva and long-term outcome in terms of CR, relapse frequency, alternative immunosuppressive agents (ISAs) use and compliance was assessed at six, 12 and 36-months’ follow-up while off levamisole therapy.

**Results:**

Leva was used in 106 patients (male 67%) for 20.4±8months during the study period. The mean age at Leva-initiation was 6.3±3 years. Eighty-three were FRNS and 23 SDNS. ESRs were 64 and 42 were LSRs. Overall, Leva was effective in maintaining short-term CR (73%) at end of Leva and it was effective in both ESRs (75%) and LSRs (69%, p=0.51) as well as in FRNS (72%) and SDNS (74%), P=0.08). Post-Leva follow-up duration was 36±31 months. Long-term Leva was effective in both ESRs and LSRs (p=0.51). Complete remission in 22,17 and 26%; infrequent relapses in 50,40 and 5% and alternate ISAs use in 24,39 and 55% at six,12 and 36 months follow-up respectively.

**Conclusion:**

Levamisole was effective in maintaining short-term and long-term remission in early and late initial steroid responsive nephrotic syndrome.

## INTRODUCTION

Nephrotic syndrome is one of the most common glomerular diseases in children. It is characterized by edema, proteinuria, and hypoalbuminemia.^1^ Majority (80-90%) of patients show complete remission (CR) within four weeks of daily oral prednisolone (OP) during initial episode. However, relapses and remissions are common, require multiple steroid courses. These repeated steroid courses are associated with long-term steroid toxicity.^2^ To avoid steroid toxicity, various immunosuppressive agents (ISAs) such as levamisole (Leva), cyclophosphamide, calcineurin inhibitors, mycophenolate mofetil(MMF) and rituximab have been used.^1^-^3^ These all are also associated with adverse effects.^2^ Therefore, less harmful agent like levamisole has been used and found effective in maintaining remission.^4^

Though exact mechanisms of effectiveness of Leva in frequent relapsing (FR) and steroid dependent nephrotic syndrome (SDNS) is not known but immunomodulation occurs by shifting the Th2 immune response to a Th1 immune response and by down regulation of Th2 mediated-immune response. Recently, it has been found that Leva also directly acts on podocyte by inducing glucocorticoid receptor (GR) signalling pathway.^5^,^6^

The rapidity of response to OP in initial episode has been considered as an important prognostic indicator in steroid sensitive nephrotic syndrome.^7^ The comparative effectiveness of Leva in ESRs and LSRs has not been studied before, although the role of levamisole in maintaining remission in FR and SDNS has been proven by many studies.^8^, ^9^ The objectives of the study were to determine the short term and long-term outcome of Leva in terms of CR, change in relapse frequency, need of ISAs in early and late steroid responders during first episode of NS.

## METHODS

This was a retrospective observational single centre study carried out from January-August 2023 on patients with FR and SDNS who received Leva as steroid-sparing agent in the Department of Pediatric Nephrology, at The Kidney Centre Postgraduate Training Institute from January 2012 to January 2022.

### Ethical Approval:

The Institutional Ethical Review Committee approval was taken (Ref#: 155-PEDNEPH-012023-Exemption; date: January 19, 2023) before embarking on study. The informed consent from individual participant and or parents was not required since it was a retrospective medical record review.

### Study criteria:

All children aged two-fourteen years with FRNS and SDNS who received Leva during the study period and had follow-up of at least six months after end of Leva-therapy were included. Patients with steroid-resistant NS, congenital NS, secondary NS, and those who received other ISA before Leva –treatment were excluded.

### Treatment protocol:

Treatment of first episode of NS during initial period of study (2012-2017) was daily OP at 60 mg/m^2^ /day for four-six weeks followed by alternate day (AD) 40 mg/m^2^ for four weeks then slow tapering over three -six months and in second period (2018-2023), daily OP at 60mg/m^2^/day for four -six weeks followed by 40 mg/m^2^/ on AD for eight weeks. Relapses were treated with daily OP 60mg/m^2^/day for two weeks followed by 40 mg/m^2^ on AD for four weeks during both study periods. For this study, we categorized patients as ESR or LSR as per operational definitions based on 2 weeks follow up spot urine protein CR ratio (suPCR) during first episode of NS. Leva was used in all patients after induction of remission with daily OP. Leva (Ketress 40mg/tablet ICI Pak) was used in all patients, in first five years, at 2.5 mg /kg as single dose on AD whereas in second five-years, it was used as 2.5 mg /kg as daily single dose.

**Fig.1:**
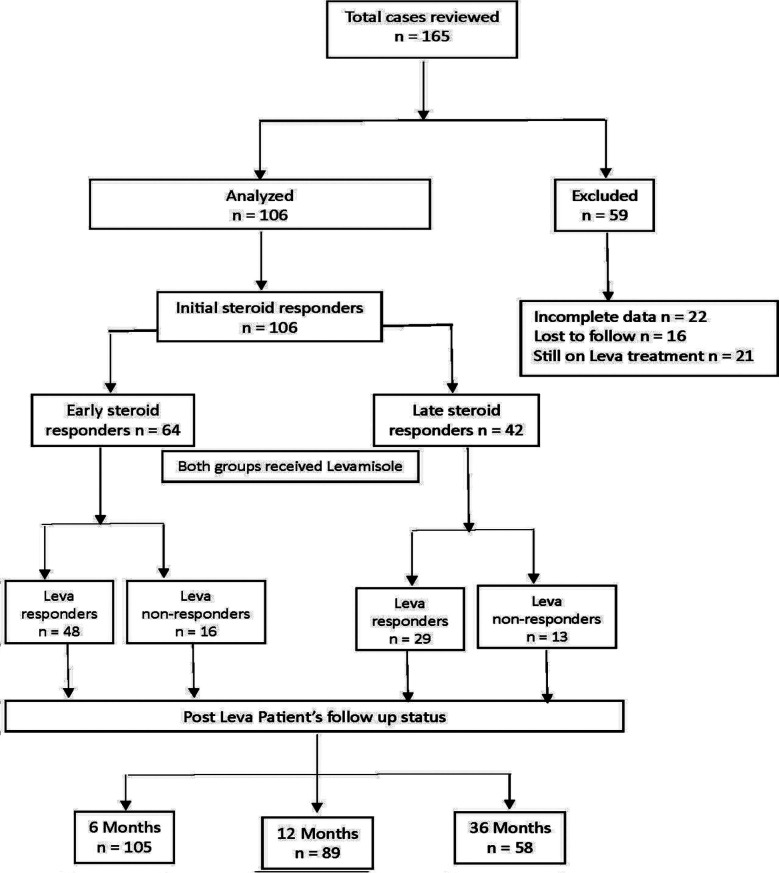
Flow Chart.

In early study period, steroids were tapered more slowly and continued throughout Leva therapy at minimum maintenance dose (0.25mg /kg) on AD whereas in subsequent study period, steroid were tapered more rapidly and were stopped at twelve months. All Leva non-responders were treated with cyclophosphamide and other ISAs. The short- term outcome of Leva was assessed at the completion of Leva-therapy (12- 24 months) and long -term outcome after discontinuation of Leva at six, 12 and 36 months follow up as shown in Flow diagram (Fig.1). We monitored response of therapy with suPCR and complete blood count (CBC) for disease remission and Leva adverse effects respectively at monthly interval for first three months and then at two-three months’ interval.

### Operational definitions:

NS was defined as combination of clinical edema, nephrotic range proteinuria (suPCR ≥2 or 3+ protein on dipstick), hypoalbuminemia (< 2.5G/dl) and hypercholesterolemia (> 250 mg/dl). Early steroid responder (ESR) was defined as complete remission (CR) achieved within two weeks of daily OP whereas late steroid responder (LSR) if CR achieved after two weeks but before four weeks. FRNS was defined as >two relapses within six months period of initial response or >four relapses in any 12-month’s duration whereas infrequent relapses were defined as < two relapses in six months or <four relapses in 12 months period.

SDNS was defined as > two consecutive relapses either during AD steroid therapy or within two weeks of stopping OP. Primary steroid resistant nephrotic syndrome (SRNS) was defined as persistence of edema and / proteinuria (suPCR > 2) after four-six weeks of daily OP during initial episode. Late SRNS / secondary SRNS were defined as those who behaved as steroid sensitive during initial episode and become steroid resistant after wards at any stage of disease course. Leva-responders were defined as either no relapse or less than two relapses in initial six months on Leva treatment. Leva- non –responders were defined as > two relapses during initial six months of Leva- therapy while Late Leva –non -responders as > two relapses in six months’ period at any time during Leva- therapy. Levamisole outcome was defined as short -term at the completion of Leva- therapy and long -term outcome as post -leva follow up minimum at six months and maximum at 36 months.

### Statistical analysis:

The data was collected from medical records of all included cases on predesigned proforma. The data included gender, age at disease onset and at start of Leva, base line clinical characteristics at the time of diagnosis, serum creatinine (Cr), serum albumin and suPCR. The triggering factor (asthma) for disease onset, initial response to steroid (ESRs/LSRs) and subsequent behaviour (FRNS or SDNS), short- term outcome (Leva -responder/non-responder) and long-term outcome (CR, change in relapse frequency, need of ISAs) and adverse effects if any were recorded. Data was analysed on SPSS version 21. Descriptive statistics was applied. Continuous variables were presented as mean with standard deviation (SD), while categorical variables were represented by frequency with percentages. The association between the categorical variables was assessed by Chi-square or Fisher’s exact test, while difference between the groups was analysed by Man-Whitney test. P value < 0.05 was considered as significant.

## RESULTS

The data of 106 patients were analysed and their baseline characteristics are shown in Table-I. The initial response to OP showed that 60.4% of patients were ESRs and 39.6% were LSRs. We did not find a significant association of age at disease onset, gender and history of asthma with initial steroid response; however, a significant association of hypoalbuminemia and microscopic haematuria with the initial response to steroid was observed (p≤0.05, Table-II).

**Table-I:** Baseline characteristics of children with steroid sensitive nephrotic syndrome (n=106).

Variables	N	%
***Gender***		
Male	71	67
Female	35	33
Mean age at disease onset(years) ±SD	3.7	± 2.2
Mean age at initiation of levamisole (years) ±SD	6.3	± 0.3
History of asthma/atopy	48	45.3
Microscopic hematuria	33	21.7
Mean serum creatinine (mg/dl) ±SD	0.21	± 0.2
Mean serum albumin (G/dl) ± SD	1.8	± 0.4
Spot urine protein creatinine ratio(suPCR) ±SD	7	± 3.7
Infections at disease onset	53	50
***Initial steroid response***		
Early steroid responders	64	60.4
Late steroid responders	42	39.6

**Table-II:** Association of clinical parameters with initial steroid response in nephrotic syndrome (n=106).

Variables	Early steroid responders N =64	Late steroid responders N=42	P-value
Age at diseases onset (years), mean ± SD	3.4 ± 1.8	4.2 ± 2.5	0.056
***Gender***	n (%)	n (%)	0.633
Male (n=71)	44(68.8)	27(54.3)	
Female (n= 35)	20(31.3)	15(35.7)	
Serum creatinine(mg/dl), mean ± SD	0.2 ± 0.2	0.22 ± 0.2	0.871
Serum albumin(G/dl), mean ± SD	1.8 ± 0.4	1.9 ± 0.4	0.05
Spot urine protein creatinine ratio, mean ±SD	6.7 ± 3.5	6.5 ± 3.1	0.839
***History of asthma/atopy***	n (%)	n (%)	0.228
Yes (n=48,45.3%))	32(66.7)	16(33.3)	
No (n=58, 54.7%))	32(55.2)	26(44.8)	
Microscopic hematuria			<0.001
Yes (n=23)	3(13)	20(87)	
No(n=83)	61(73.5)	22(26.5)	
Cumulative steroid dose/pt(mg), mean ± SD	17040.1±16885.3	19593.9±14249.7	0.54
Late SRNS*			0.999
Yes (n=8)	5(62.5)	3(37.5)	
No(n=98)	59(60.2)	39(39.8)	

* SRNS=Steroid resistant nephrotic syndrome.

The association of clinical and biochemical parameters with levamisole outcome are shown in Table-III. The mean duration of Leva therapy was 20.4±8 months. At the completion of Leva- therapy, majority (73%) were Leva- responders (Fig.2a). There was no significant difference in Leva-response between ESRs (75%) and LSRs (69%, p=0.51). Similarly, there was no difference in Leva –response between FRNS and SDNS (P=0.08). A better response in AD-Leva users (91%) compared to daily Leva -users (64.4%, P=0.005) was observed.

**Table-III:** Association of clinical and biochemical parameters with levamisole outcome. (n=106).

Variables	Leva responders n=77	Leva non-responders n=29	P-value
Age at disease onset (years), mean ±SD	3.6 ± 1.9	5±1.7	0.273
Age at Leva initiation (years), mean ±SD	6.3±3.0	6.2±2.8	0.98
***Gender n (%)***			0.04
Male	56(72.7)	14(48.3)	
Female	21(27.3)	15(35.7)	
Serum creatinine (mg/dl), mean ±SD	0.2 ±0.2	0.2 ± 0.2	0.283
Serum albumin (G/dl), mean ±SD	1.9 ± 0.4	1.8 ± 0.3	0.346
Spot urine protein creatinine ratio ±SD	6.4 ± 3.1	7.3 ± 3.8	0.196
***History of asthma***	***n (%)***	***n (%)***	0.62
Ye(n=48)	36(46.8)	12(41.4)	
No (n=58)	41(53.2)	17(58.6)	
***Microscopic hematuria***			0.082
Yes(n=23)	20(87)	3(13)	
No (n= 83)	57(68.67)	26(31.32)	
***Indication of levamisole***			0.08
FRNS* (n=83)	60(82.3)	23(27.7)	
SDNS* (n=23)	17(74)	6(26.)	
***Schedule of Leva use***			0.005
Alternate day (n= 33)	30(90.90)	3(9.09)	
Daily dose(n=73)	47(64.38)	26(35.61)	
Duration of Leva mean ± SD	22.7±6.29	14.1±9.0	0.02
Cumulative steroid dose /pt(mg), mean ±SD	17205.1±13842.9	20300.5±20430.5	0.38
Cumulative Leva /pt(mg), mean ±SD	29683.8±18659.7	18941.3±15009.7	0.23
***Initial response to steroid***	***n (%)***	***n (%)***	0.51
Early steroid responders n=64	48(75)	16(25)	
Late steroid responders (n=42	29(69)	13(31)	
***Biopsy findings n=30(28%)***			0.847
MCD** n=17(56.66%)	10(58.82)	7(41.17)	
FSGS**n=10(33.33%)	9(81.8)	1(11.1)	
Others(n=03(10%)	2(18.2)	1(1.1)	
***Late steroid resistance***			0.034
Yes(n=8)	3(37.5)	5(62.5)	
No(n=98)	74(75.5)	24(24.5)	

FRNS and SDNS=Frequent relapsing and steroid dependent nephrotic syndrome,

** MCD=minimal change disease, FSGS=focal segmental glomerulosclerosis, pt=patient.

**Fig.2A:**
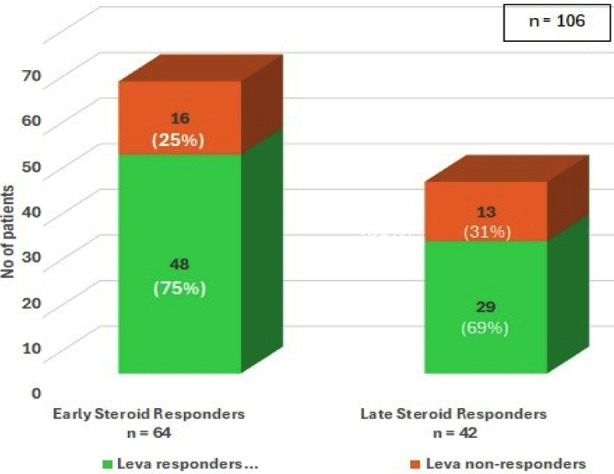
short term outcome of levamisole at end of treatment in early versus late steroid responders.

The mean post- Leva follow- up duration was 36+31months. Long -term post Leva -outcome at follow up of six (n=105), 12 (n=89) and 36 months (n=58) is shown in Table-IV and Fig.2b. There was no difference in long-term outcome of Leva in ESRs compared to LSRs (p=0.51) at all three respective follow up periods. This table shows long -term outcome with respect to nephrotic status like CR, relapse frequency, alternate ISAs use and compliance to follow-up. Complete remission of proteinuria was observed in 22, 17 and 26% at respective periods. Alternative ISAs were used in 24, 39 and 55% at respective follow up. However, use of ISAs was delayed in Leva- responders. We observed reversible leukopenia in one case.

**Table-IV:** Long-term outcome of levamisole in steroid sensitive nephrotic syndrome (N= 106).

Levamisole outcome status		Post levamisole follow-up duration		

Outcome at end of levamisole		06 months	12 months	36 months
	n (%)	105 (99)	89 (84)	58 (54.7)
Leva -responders	77(73)	76(72.38)	64(72)	44(75.86)
Leva non-responders	29(27)	29(27.61	25(28)	14(24.13)
Post levamisole outcome				
Complete remission		23(22)	15(16.85)	15(25.86)
Infrequent relapsers		53(50.47)	32(39.9)	3(5.17)
Other immunosuppressive agents		25(23.80)	35(39.30)	32(55.17)
Lost to follow		4(3.80)	7(7.86)	8(13.7)

**Fig.2B:**
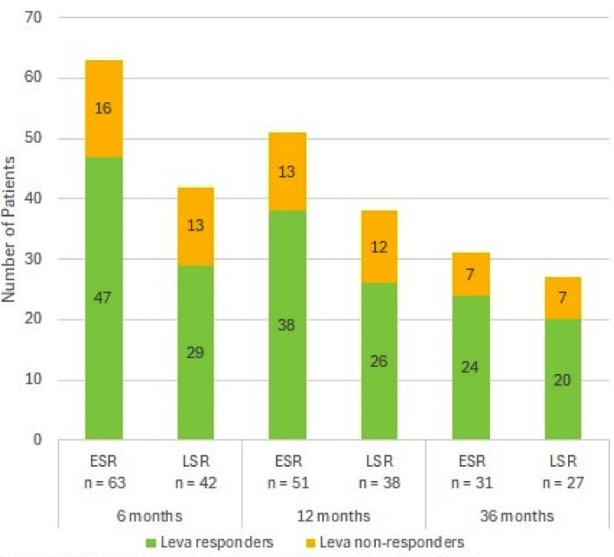
Long-term outcome of levamisole in early versus late steroid responders. ESR=early steroid responder, LSR=late steroid responders.

## DISCUSSION

In our study, majority of patients(60.4%) were ESRs during first episode of nephrotic syndrome, which is consistent with the other studies.^7^,^10^ A recent study from our neighbouring country showed a similar response to steroid therapy (65.3%), though slight variation in categorization exists.^11^ Our finding of 73% short -term effectiveness of Leva is consistent with our earlier study where we found its effectiveness in 76.4%.^8^ In other studies, the reported effectiveness of Leva is 80-88%.^4^,^9^ The response of Leva in our study in maintaining relapse free remission in ESRs (75%) and in LSRs (69%) was not significantly different (P=0.51, Table-III, Fig.2a).This is in contrasts with a recent study in which poor Leva response in LSRs has been reported.^11^

Levamisole has been used historically on alternate day basis and also recent IPNA clinical practice guidelines recommend alternate day use but it has been used on daily basis in many studies and found better response.^1^,^4^,^12^,^13^ A better response of Leva (Table-III) when used on AD compared to daily basis (P=.005) is an unusual, since idea of daily use is to get better response as shown in other studies.^14^,^15^ The possible explanation of this better response in our study may be slow tapering of steroid along with AD- Leva during the early period compared to later period of the study. In the present cohort, majority were FRNS (78%) and there was no significant difference (p=08) in short- term efficacy of Leva in FRNS compared to SDNS ((83 vs 74%). This is relatively in contrast to our earlier study and to other studies.^8^,^9^,^12^

Long- term Leva-outcome (Fig.2b) did not show significant difference in ESRs versus LSRs ((p=0.51) at six, 12 and 36 months follow- ups. Long - term CR was observed in 22,17and 26% at respective follow- ups and infrequent relapses were observed in 50, 40 and 5%. Similar long-term effects have been reported by other studies.^16^-^19^ Boyer O and Elmas AT reported post leva outcome at 12 months and found effectiveness in 67.64 and 62% respectively.^17^,^18^ Similarly, Sumegi V^19^ et al has shown long term effectiveness at 24 months post -leva follow up as 82.35%. Thus, we may claim that this is fist study showing long-term outcome after 36 months of post- leva follow- up. Despite these beneficial effects, some patients (24%,38% and 55%) required alternative ISAs at different follow up periods in both leva-responders and non-responders though ISAs were required early in Leva non-responders (Table-IV). Similar proportion of alternate ISAs use has been reported in other studies.^11^,^12^

Levamisole efficacy in FRNS has been compared using AD and daily dose in RCT with MMF and found similar efficacy in maintaining CR 40.8 vs 34.2% and 65% vs 56% respectively.^20^,^21^ Levamisole has also been compared with MMF in patients with SDNS after cyclosporine withdrawal and found not inferior to MMF.^22^ A reversible leukopenia in our case is lower than that reported in other studies.^4^,^11^,^13^

### Limitation:

The retrospective nature and the diversity of corticosteroid and Leva- protocols for initial episode and subsequently in two different eras of study are the major limitations. However, Leva-outcome in a large cohort in ESRs and LSRs as well as a long post-Leva follow- up are the main strengths of our study.

## CONCLUSION

Levamisole was equally effective in maintaining short and long- term remission in ESR and LSRs children. It maintained either complete remission or changed behaviour from frequent relapsing/steroid dependency to infrequent relapsing nephrotic syndrome. Thereby, levamisole use prevented children from steroid toxicity and delayed ISAs use in steroid sensitive nephrotic syndrome.

### Recommendation

Use of Leva in SSNS in developing country may avoid alternative ISAs use and associated high-cost burden and toxicity. However, further studies are needed to find out its optimal duration and steroid withdrawal protocol during Leva-therapy.

## List of abbreviations:

Levamisole (Leva), Nephrotic syndrome (NS), Early steroid responder (ESR), Late steroid responder (LSR), Frequent relapsing nephrotic syndrome (FRNS), Steroid dependent nephrotic syndrome (SDNS), Oral prednisolone (OP), Alternate day (AD), Spot urine protein creatinine ratio (suPCR), Complete remission (CR), Immunosuppressive agents (ISAs).
